# The European Universities initiative: between status hierarchies and inclusion

**DOI:** 10.1007/s10734-023-01167-w

**Published:** 2024-01-05

**Authors:** Agata A. Lambrechts, Marco Cavallaro, Benedetto Lepori

**Affiliations:** Università della Svizzera italiana, Institute of Communication and Public Policy, Lugano, Switzerland

**Keywords:** European Universities initiative, University alliances, Transnational alliances, Institutional cooperation, European policy, Higher education

## Abstract

Using a dataset of higher education institutional alliances within the framework of the European University initiative (EUi), we test empirically whether the policy-defined goal of a relative balance between *excellence* and *inclusiveness* within the scheme has been achieved. Specifically, we provide a descriptive and analytical account of the diversity of the higher education institutions (HEIs) participating in the EUi, the composition of—as well as the mechanisms behind—the formation of individual alliances. We observe that alliance formation activated the deep sociological mechanisms of hierarchisation, with the alliances largely reproducing the existing *hierarchy of European HEIs*. Specifically, we argue that the global-level stratification hierarchy cast by rankings influences the participation of individual institutions and—although to a more limited extent—the formation/structure of the alliances. Further, we demonstrate that the EUi has strengthened existing ties since most alliances thus far have built on existing forms of collaboration. However, we also show empirically that some of the distinctive policy design measures, namely the requirement for broad geographical coverage and generically framed rules for participation, as well as opening the initiative to new alliances and encouraging enlargement of the existing ones, have generated opportunities for involvement of the lower-status institutions. This broadened the scope of the EUi beyond the core of top-ranked research universities located in the knowledge production centres of Europe. We suggest that these observations may have important implications for how the intended extension of the EUi may be implemented in the future.

**Supplementary Information:**

The online version contains supplementary material available at 10.1007/s10734-023-01167-w.

## Introduction

Like other parts of the world, Europe is facing major challenges, and “strong, inter-connected higher education institutions” (HEIs) (European Commission, 2022) have been identified as the key instrument in “shaping sustainable and resilient economies, and in making the European Union greener, more inclusive and more digital” (European Commission, 2022). To enable HEIs to contribute to these objectives, the European Commission (EC), with support from the European Council and the European Union (EU) Member States (MS), has set up several ambitious flagships. The *European Universities initiative* (hereafter the EUi), supporting the creation of strategic transnational alliances of HEIs, has taken centre stage.

The European Commission sets out the overarching aims for the initiative and provides evaluation criteria for the selection and funding of new alliances. Within this framework, the EUi encourages the creation of bottom-up networks with broadly defined boundaries. The applications are open to all types of institutions, from traditional universities to universities of applied sciences, business schools, academies of arts, etc. The alliances must include a minimum of three HEIs, each holding a valid Erasmus Charter for Higher Education, located in either an EU MS or a third country associated with the Erasmus+ programme, with partners coming from different parts of Europe^1^. These broad criteria construct, at least in theory, a large potential tie pool of all types of HEIs within a wide geographical area.

The Council conclusions on the European Universities initiative from May 2021 asserted that the intention for the scheme was to strike “the right balance between quality and excellence, on the one hand, and inclusive and equitable geographical coverage on the other” (Council of the European Union, 2021, para. 20), thus addressing head on the perhaps most often discussed question concerning the EUi thus far, i.e. the relative importance of *inclusiveness and cohesion* and *excellence* within the scheme (Birk, 2019; Gunn, 2020; Rensimer & Brooks, 2023). In his early work on alliances, Gunn (2020), for example, envisaged two potential scenarios for the EUi: an “inclusive [one], as a broader range of institutions have a place where they can find compatible partners”, or one which “may comprise of elite institutions, furthering the stratification of higher education in Europe” (p. 24). Three years on, and a few more rounds of funding, while the early evaluations of the initiative are still ongoing, little empirical evidence has been presented to aid our understanding of whether the scales have tipped towards either scenario.

To advance the literature, in this study, we examined the institutional and geographical diversity of HEIs participating in the EUi and the composition of individual alliances to assess the balance between excellence and inclusiveness achieved thus far. We focused on two research questions: first, has a balance been found between the policy goal of broad participation of different types of HEIs and the lasting role of elite institutions, such as those included in the upper echelons of the global rankings? Second, to what extent does this achieve wide European coverage and integrate HEIs from “new” EU Member States, which are less represented in international rankings?

To address these questions, we rely on different strands of literature, including the literature on network formation and the role of status in shaping collaboration networks (Collet & Philippe, 2014; Podolny, 1994), the literature on the formation of alliances specifically in higher education (Brankovic, 2018b; Dusdal et al., 2021; Gunn & Mintrom, 2013; Zapp et al., 2021), and the literature on vertical stratification in higher education (Bleiklie, 2003; Jappe & Heinze, 2023; Marginson, 2013).

Our findings suggest that status hierarchies have indeed played a central role in constructing the core of EUi’s membership and that, at least initially, alliances were built primarily by HEIs with pre-existing institutional relationships, yet our data suggest some level of opening of alliances to non-ranked HEIs. Our data also show that, when combined with the EC evaluation criterion of the geographical diversity of alliances and with a specific mechanism to enlarge existing alliances, this led to an extension of this “network of networks” (Gunn, 2020, p. 18) beyond the institutions at the top of the status hierarchy and to the achievement of good geographical coverage of EU MSs and other European countries. Our main conclusion is, therefore, that the design of the EUi was indeed conducive to moderating the impact of status hierarchies and achieving some level of EU-wide cohesion.

## Theoretical framework

Status, that is the “socially constructed, intersubjectively agreed-upon and accepted ordering or ranking of (...) organisations (...) in a social system” (Washington & Zajac, 2005, p. 284), is a major organising factor of higher education worldwide. We note that status differs from *reputation*, reflecting the perceived quality based on recent past performance. *Status*, on the other hand, is a more medium-term stakeholder evaluation (Alajoutsijärvi et al., 2022) “conferred upon organisations based on the extent to which they conform to particular desirable attributes” (Patterson et al., 2014) relative to their peers—in the case of HEIs, mainly the high—or “excellent”—research performance.

Although concerns about organisational reputation and status have been present in the field for some time (Clark, 1978; Bleiklie, 2003), these have been augmented by national and international developments (namely the introduction of global rankings and national excellence initiatives) to the extent that, as noted by Marginson (2011), “[i]t is [now] often remarked that status is more important to universities than money” (p. 31). HEIs engage in positional competition (Marginson, 2013), seeking ranks, awards, and labels of reputation and/or status, which can benefit in particular the high-status institutions, sending important signals to stakeholders (Podolny, 2010), and thus preceding or even replacing future judgements of quality by a wide audience of policymakers, prospective students, academic personnel, the general public, and funders (Jappe & Heinze, 2023; Wilbers & Brankovic, 2021). Funding is increasingly awarded predominantly to the research-active (already) resource-rich “elite” HEI, in turn increasing their research outputs and contributing to their international standing—an exemplification of the so-called Matthew cumulative effect (Lepori et al., 2019). This leads to increasing and entrenchment of stratification of the HE sector in Europe (Lepori et al., 2015; Teichler, 2007), as well as growing regional inequalities, with the status of HEIs in the wealthier regions increasing while the poorer regions are left with the weaker institutions (Huisman & van Vught, 2009; Quaglio et al., 2020).

As discussed next, existing studies suggest that status plays an important role also in the process of network formation between organisations, including those involving HEIs.

### The role of status (and pre-existing ties) in networks formation

In management and organisational studies, there is a rich body of literature on inter-firm alliance formation, including both considerations for entering the alliances, such as reducing costs, knowledge exchange, increasing legitimacy and credibility, and accessing new markets (e.g. Anand & Khanna, 2000; Li & Ferreira, 2008) and the basis for the formation of alliances. One of the most commonly cited mechanisms for tie formation is the attribute-based homophily (Siciliano et al., 2021), that is the tendency for alliances to be formed between organisations that are similar in terms of certain attributes or characteristics such as size, age, geography, and social environment, or clients’ demographics (e.g. Franco & Haase, 2012; Kim & Higgins, 2007).

Specifically, previous research has evidenced that social relationships, including those between organisations, tend to be linked to *status homophily* (Burris, 2004): social relationships are both powerful markers of status and a mediator as organisations associate with others with similar positions in the status hierarchy to maintain or improve their own standing reputation and status (Chung et al., 2000; Podolny, 1994), which in turn may allow them to influence the rules governing future competition for status and resources alike (Piazza & Castellucci, 2014; Podolny, 2010).

Other studies, however, have demonstrated that under specific circumstances, this natural tendency to form ties with similar organisations can be overridden (Castellucci & Ertug, 2010; Shipilov et al., 2011). Findings from Collet and Philippe (2014) suggest that market conditions can influence the “use and interpretations of the heuristics [the organisations] rely upon” (p. 424) and thus the type of relationships initiated by organisations. The authors have shown that organisations that do not want to miss out on high-potential opportunities focus on upside risks, paying less attention to status cues, and initiating heterophilous ties, in particular during up market.

In addition to attributes such as size or geography and status, much research also suggests that organisations are more likely to form alliances with actors with whom they have pre-existing ties, organisations they trust, and with whom they share a history of a rich exchange of information (Anand & Khanna, 2000; Gulati, 1995). Further, previously unconnected organisations are more likely to form ties if they both have ties to a common third-party organisation (Gulati, 1995). The previous direct or indirect ties foster information sharing in the new alliance, reduce the search costs, and mitigate the risks associated with opportunism (Gulati, 1995; Gulati & Gargiulo, 1999).

### Formation of alliances in higher education

Participation in institutional alliances, such as strategic partnerships, networks, and associations, has become widespread also in higher education (Marques et al., 2022). Beyond their practical functions as platforms for cooperation in education and research, building capacity, or establishing a collective voice to represent the interests of member institutions (Beerkens, 2002; Brankovic, 2018b; Sandström & Weimer, 2016), alliances play a core role in demarcating groups of “similar” institutions, such as “research universities” or “universities of applied sciences” (Brankovic, 2018a). As in other sectors, participating in HE alliances allows mutual learning and sharing of key resources, including connections with other actors, thereby generating strategic advantages for partners (Gunn & Mintrom, 2013) and enhancing their competitiveness (Brankovic, 2018b; Hüther & Krücken, 2016).

The basis for alliance formations and their compositions varies. Some alliances are more exclusive, with membership based on geography, mission, or status (Brankovic, 2018b), and subject to strong control by their members (Beerkens, 2018). Others, such as university association, are more inclusive and open to a wide range of organisations located both in the centres and peripheries of the field (Dusdal et al., 2021). While lower-status HEIs may be drawn to join these more inclusive alliances in order to improve their standing in the field (Zapp et al., 2021), the higher-status HEIs may choose to join alliances with similar-status partners to both enact and enhance their status in the field (Brankovic, 2018b), as it affords HEIs more legitimacy and, thus, resource stability (Brankovic, 2018a).

In accordance with the above, we expected that status and pre-existing network ties are likely to play a central role in EUi participation (see also Charret & Chankseliani, 2022). However, the European Universities initiative is distinctive from other HE intra-organisational partnerships in its aim of creating a “network of networks” that is a large number of unique alliances with exclusive membership that are “united through their membership of a top-down strategic scheme with common overarching aims and objectives” (Gunn, 2020, p. 18)—the European Union’s selection of alliances is conditioned by policy goals, such as inclusion and geographical cohesion^2^, which are likely to lead to a broad representation of HEIs’ institutional types and to extend (previous) networks to the whole of Europe.

To what extent this goal of inclusiveness allows overcoming the status homophily tendency in EUi formation is a core question addressed in this paper.

## Data and method

### Sample

Our sample is composed of the 44 alliances funded under 2019, 2020, and 2022 calls, involving a total of 356^3^ HEIs in 33 countries that participated in the initiative as of spring 2023.

As a result of 2019, and 2020 pilot calls with 116 applications submitted, 41 alliances involving 282 HEIs were established. In the summer of 2022, following the first call for long-term support, attracting 52 applications, 16 existing alliances founded under the first “wave” of the pilot received confirmation of continued funding. All have been joined by new members, with only a couple of HEIs leaving the alliances and one moving from one to another. Further, four new alliances received backing from the EC.

Our analysis included these 44 alliances and their participating HEIs^4^. The number of HEIs in different analysed dimensions may slightly vary, depending on data availability.

This sample was compared with the population of HEIs included in the European Tertiary Education Register (ETER, https://www.eter-project.eu/)—the European-level database on higher education providing a reference list of HEIs Europe and quantitative data at the institutional level on core HEIs’ activities and outputs, such as educational activities (students and graduates), research activities (PhD researchers), and personnel and finances (Lepori et al., 2023). ETER provides largely complete coverage of HEIs awarding degrees at the bachelor, master, and PhD levels in the concerned countries.

This population included 2801 HEIs in 33 European countries (EU-27, Iceland, Norway, Switzerland, Serbia, UK, and Turkey).

### Data

We derived from ETER a set of variables that describe some core characteristics of HEIs to compare those HEIs participating in alliances with the overall population of HEIs in Europe as described by ETER (Huisman et al., 2015) as in Table 1. These include the legal right of awarding a PhD, as the main distinction between university and non-university sectors, a measure of organisational size based on academic personnel, a (size-normalised) measure of educational intensity, and two complementary measures of research orientation (based on PhD students and publications), as well as a measure of orientation towards science and technology (based on the distribution of students). Given the international character of alliances, we finally include two complementary measures of internationality, i.e. the share of mobile students and the (size-normalised) number of incoming Erasmus students. The data are derived from ETER, except publication data, which are derived from the RISIS research data infrastructure (https://www.risis2.eu/).

**Table 1:** Core institutional characteristics variables and indicators

Variable	Definitions and remarks	Source
PhD awarding	Legal right to award a PhD	ETER
Organisational size	Number of academic personnel in full-time equivalents	ETER
Educational intensity	Number of students (levels 5 to 7 of the International Standard Classification of Educational Degrees (ISCED)*	ETER
PhD intensity	Number of PhD (ISCED 8) students*	ETER
Publication intensity	Number of scientific publications in the Web of Science (based on the definitions adopted for the Leiden ranking)*	RISIS-OrgReg
International orientation	Erasmus IN: number of incoming Erasmus+ students (ISCED 5–7)*	ETER
	Share of foreign students: share of ISCED 5–7 students educated abroad before starting their studies	ETER
STEM orientation	Share of ISCED 5–7 students enrolled in STEM disciplines	ETER

*Normalised by organisational size

Data refer to the academic year 2019/2020, except where only earlier data was available at the extraction time (with 2016/2017 data used for the Czech Republic, Cyprus, and Bulgaria).

For the analysis of the relationships between HEIs and global status hierarchy, we have utilised the ranking positions in the Academic Ranking of World Universities in 2021 (https://www.shanghairanking.com/); ARWU is one of the oldest, most established, and most visible rankings that have been associated with global status and stratification of HEI systems (Cantwell & Taylor, 2013; Hazelkorn, 2015). Regardless of the specific factors they measure, or the methodologies employed, rankings have become ubiquitous signals of status positioning accepted by prospective students, faculty members, alumni, employers, and funding agencies in higher education (Espeland & Sauder, 2007) and thereby contributing to the stratification of the HEI system.

Finally, to analyse the role of pre-existing ties on EUi participation, we have resorted to quantitative and qualitative data. First, we examined whether alliances have been preferentially formed among HEIs cooperating in EU-FP programmes using data from CORDIS (https://data.europa.eu/en) and exchanging of Erasmus+ students (Gadár et al., 2020). The EU-FP collaborations have been chosen as the main instruments for implementing common European scientific and innovation policy, with the largest budget available for supporting research in European HEIs. The Erasmus+ database was selected as the most comprehensive publicly available record of HEI educational collaboration. Both datasets cover the years 2014 to 2020.

In addition, we have created a dataset of EUi members’ participation in other institutional partnerships and networks preceding the EUi using information collected from different sources: information on existing well-known associations, such as the League of European Research Universities and the Coimbra Group, a hand search through various websites, and a Google search using queries such as “university OR higher education” and “association OR partnership OR network OR alliance OR consortium”. Whole-organisation networks were included in the sample, while lower-level collaborations (i.e. at the research group, department, or faculty level) were excluded. We have further compared our list of identified networks with a database of university associations created (and kindly shared) by Jelena Brankovic for her study on meta-organisations (last updated in 2017; Brankovic, 2018b).

### Analysis

The statistical analyses were performed using a standard software package (Stata, version 13.1).

First, we compared the median characteristics of HEIs participating in EUi with those of the whole population of HEIs delivering at least a bachelor’s degree in European countries as provided by ETER, to test whether HEIs participating in EUi are systematically different in terms of core institutional attributes (Table 1). The significance of these differences was tested using the Kruskal-Wallis test for the equality of medians, a non-parametric test that is robust against the non-normality of distributions. We also compared the “old” HEIs participating in the EUi (2019 and 2020 calls) with the “new” ones (2022 call) to ascertain whether the further enlargement includes HEIs with different characteristics.

Second, we examined whether ranking position (i.e. status marker) is the central predictor for participation in the EUi and the basis of alliance formation. It has been operationalised based on the university rankings provided by ARWU (2021 ranking) for both EUi-participating and non-participating European HEIs. The indicator ranges from 1–100 to 1000+ (HEIs in the 1000+ bracket are referred to as “unranked” institutions). We have used descriptive statistics (percentages) to describe the relationship between status and participation in the EUi and to characterise the individual alliances in relation to their members’ status.

Third, we analysed geographical patterns of participation, looking specifically at the intensity of participation with respect to the size of the national higher education system, as measured by the number of tertiary education students derived from Eurostat. Given the debate on EU cohesion and extension, we specifically focus on participation patterns of the HEIs in “new” EU MSs and candidate states (the so-called EU13 countries). Since ranked HEIs are concentrated in the old EU Member States, we also check whether there are systematic patterns by country in their participation in EUi.

Finally, we examined whether alliances have been preferentially formed among HEIs with pre-existing ties. To this aim, we have, first, computed the number of joint participations to EU-FP projects and the number of Erasmus students exchanged between each pair of HEIs participating in the alliances in the period 2014–2020, both as absolute numbers and normalised by the number of students enrolled in the HEIs participating in the alliance. Second, we analysed EUi members’ joint/overlapping participation in other institutional partnerships and networks preceding the EUi.

## Findings

### Descriptive analysis

As illustrated in Table 2, there are significant differences between the HEIs participating in the EUi and those which do not in terms of *core institutional characteristics*.

**Table 2:** Factors associated with institutional participation in the EUi: European-level analysis. Median by group

	EUi old	EUi new	Non-EUi
N. of institutions	282	74	2445
PhD awarding	212	50	665***
Organisational size	1589.58	819.04**	175.62***
Education intensity	14.23	15.03**	18.03***
PhD intensity (only PhD awarding HEIs)	0.0373	0.0217**	0.0173***
Publication intensity	0.2932	0.1770***	0.0000***
International orientation: Erasmus IN	0.3654	0.3855	0.2038***
International orientation: share of foreign students	0.1080	0.0739*	0.0726***
STEM orientation	0.2558	0.2569	0.0741***

KWallis test of equality of medians (new/old EUi members, all EUi members/non-EUi)

Differences are significant at 0.001 (***), 0.01 (**), and 0.05 (*)

EUi old-member institutions before October/November 2022; New EUi—members that joined in late 2022 as new members of existing alliances or as members of the newly established alliances

Participating HEIs have overall substantially more resources in terms of academic personnel and are more oriented towards research than HEIs in the whole population. They have a stronger international profile—allied institutions have a higher share of internationally mobile degree students and receive substantially more students through Erasmus+ than the non-participating institutions.

Some 92% of the HEIs participating in the alliances have the right to award PhDs, against only 48% in the whole ETER population; therefore, despite claims of including all types of HEIs, EUi participation is by and large limited to the university sector. While the member institutions constitute less than 10% of the HEIs within the ETER perimeter, they enrol a quarter (25%) of ISCED 6 and 7 (i.e. bachelor’s and master’s) students, and close to half (44%) of all doctoral students in the ETER population.

This descriptive analysis, therefore, shows that HEIs participating in the alliances are mostly large and research-oriented universities.

There are also some statistically significant differences between the “old” and the “new” EUi member institutions; the new members are significantly smaller and less research-oriented than the old members, suggesting that the successive enlargement of alliances in 2022 extended participation beyond the most research-intensive universities.

### Global status and formation of alliances

Turning now to status, the general picture emerging from the analysis is that, indeed, high-status HEIs, as observed through the lenses of international rankings, are overrepresented in EUi, particularly among the “old” members, but there is also significant participation on non-ranked HEIs, particularly among the “new” members.

**Fig. 1:**
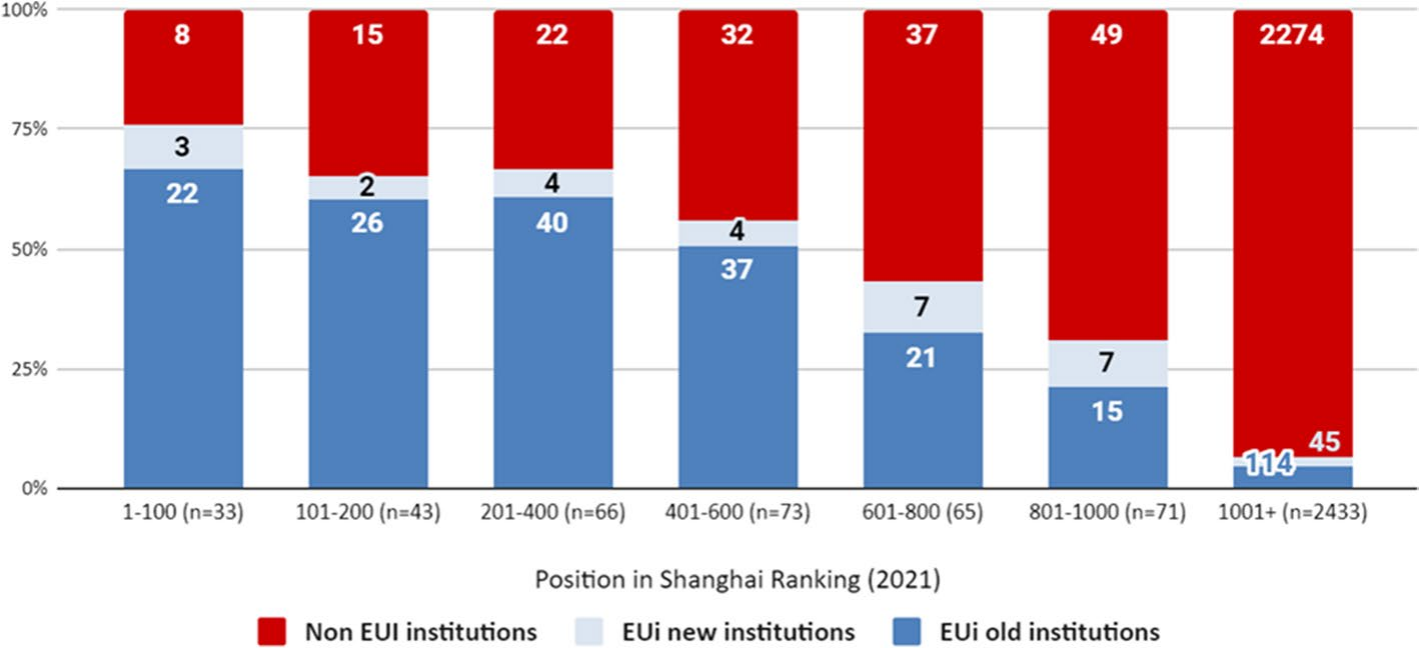
Share of EUi participants and non-participants per ARWU Shanghai Ranking (2021 ranking) position

Some 53% of all EUi members are ranked in the top 1000 institutions in the world in the ARWU Shanghai Ranking 2021. EUi already includes three-quarters of the top-ranked European institutions (ranked between 1 and 100) and some two-thirds of those in the upper brackets (ranked between 101 and 500) (Fig. 1). However, new participants in the EUi (both members joining existing alliances and members of the four new alliances) are more likely to be unranked institutions (61% of all new participations). In fact, only two of the new members are ranked in the top 100. Notably, some of the alliances, which earlier included only higher-ranking HEIs, have expanded their membership to include unranked institutions.

Therefore, most top-ranked European universities participate in the EUi, but the diversity of new members grows as the number of top-ranked universities, eligible but not yet participating in the initiative, diminishes.

**Fig. 2:**
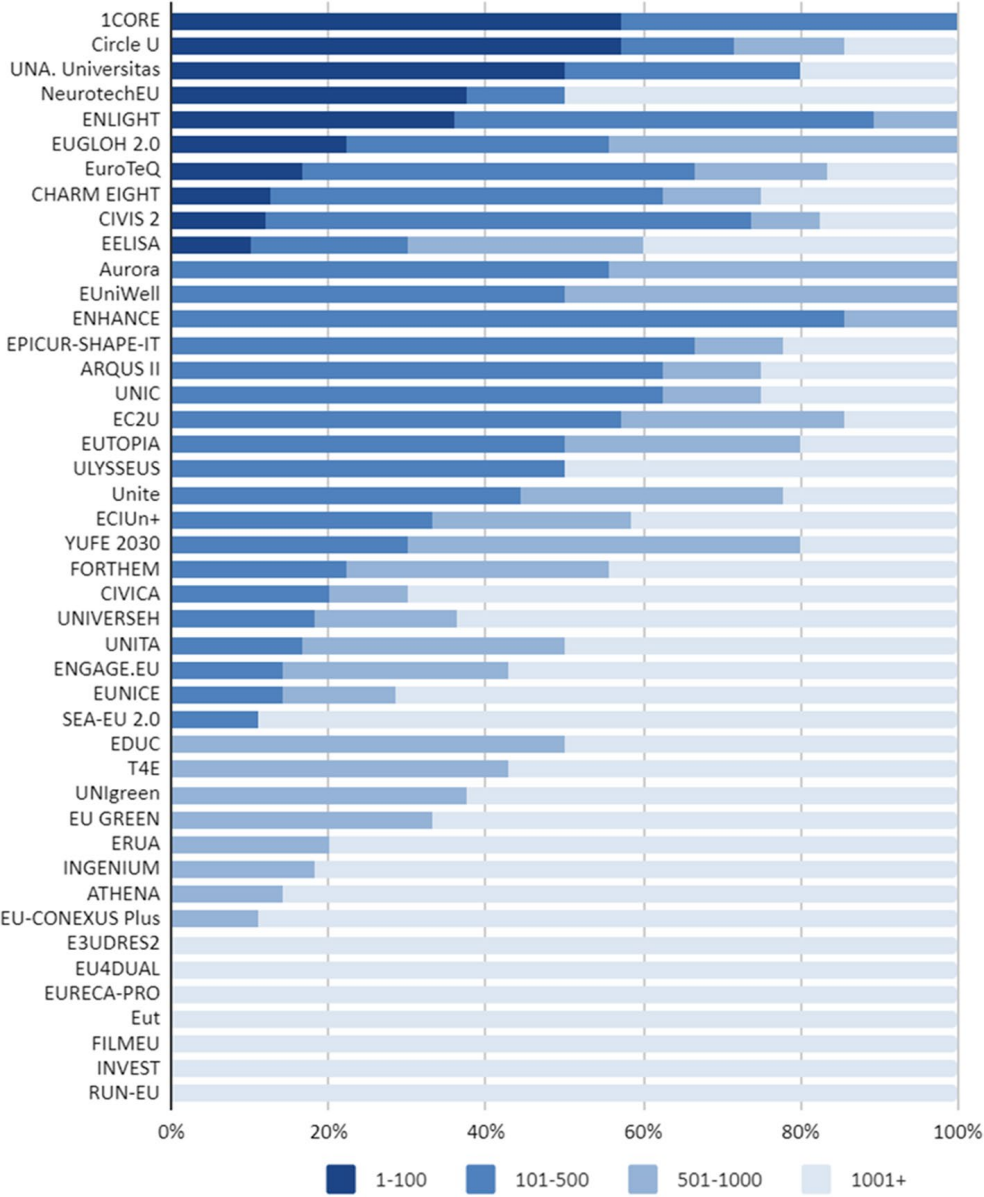
Composition of EUi alliances by ranking position (ARWU Shanghai Ranking 2021) of member institutions

As illustrated by Fig. 2, around half of the alliances included a core of ranked HEIs. However, the composition of individual alliances is somewhat varied: only 12 alliances included solely members from neighbouring status strata, while 25 alliances included a mix of institutions from different status strata (of those, only 6 do not include any unranked institutions).

Finally, only seven alliances are composed entirely of unranked HEIs. Most of these are composed of specialised HEIs on a specific topic of interest: films for FILMEU, sustainable regional development for INVEST, regional development for RUN-EU, and responsible consumption and production for EURECA-PRO and/or by institutions of the same type: E3UDRES2 and RUN-EU are primarily made up of universities of applied sciences; EUT includes only technical universities/universities of technology (and one university of applied sciences); FILMEU only universities of arts/humanities. Their number is, however, low when compared with the more broad-scope alliances based on global status hierarchies (Fig. 2).

Therefore, our data suggests that, indeed, the rankings-based global hierarchy plays a role in influencing the participation of individual HEIs and the composition/structure of the alliances within the EUi; however, data also suggest a significant degree of mixing between ranked and non-ranked HEIs.

To understand why this might be, we further considered the relationship between status position and the geographical location of the participating institutions.

### Geographical patterns and status

The number of *participations by country* shows a relatively wide coverage of European macro-regions (as shown in the Online Resource 1). A sizable proportion—some 40% of participating HEIs come from only four countries: Germany (12%), France (20%), Spain (9%), and Italy (8%), with 19 of the alliances coordinated by an institution in Germany (8) or France (11).

**Fig. 3:**
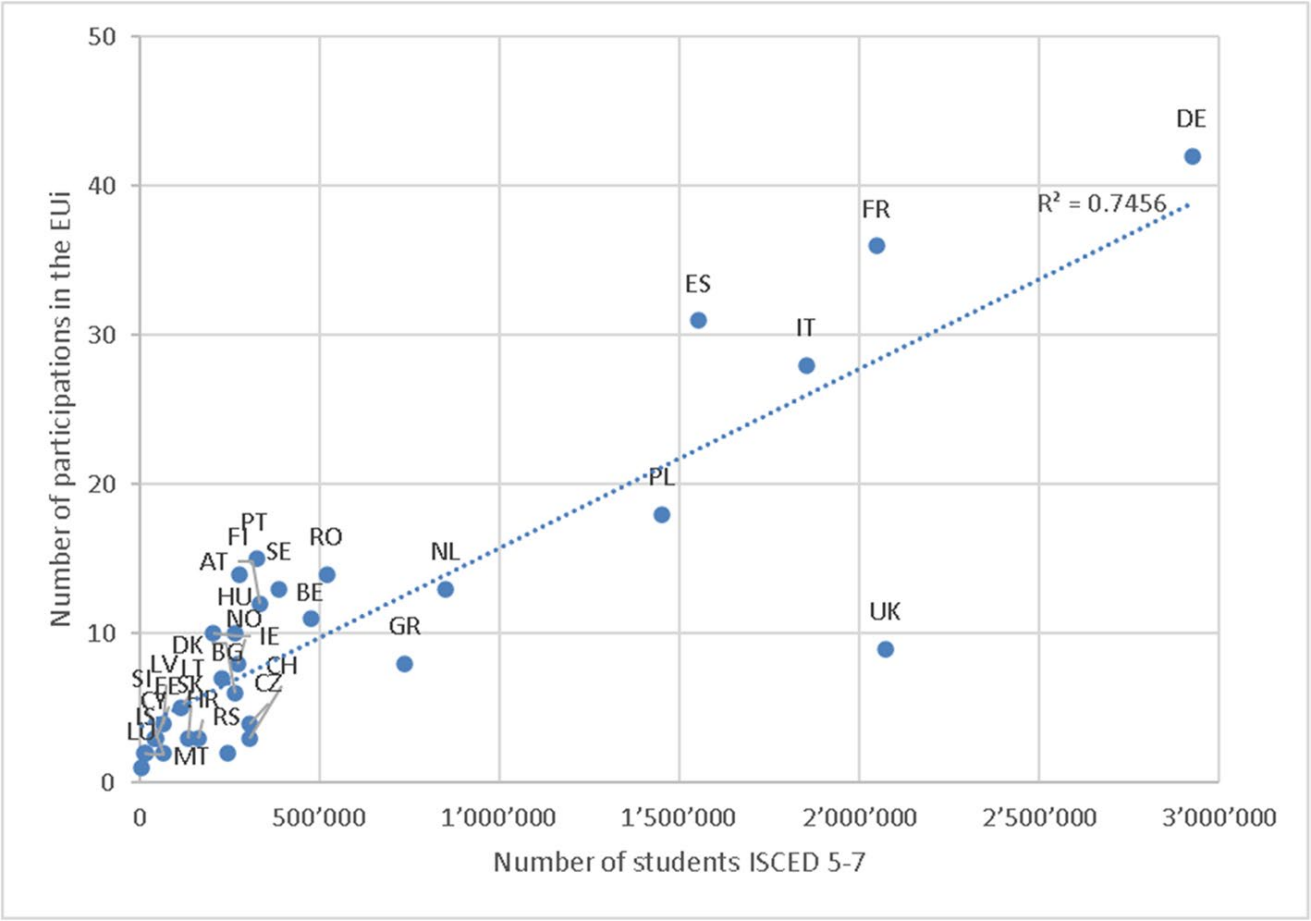
Participation in the European Universities initiative by country relative to the higher education system size (ISCED 5–7, Eurostat 2018). *R*-squared = 0.7455

However, these figures are less striking if we consider these countries’ relative HE system size measured by the number of tertiary education students derived from Eurostat (2018 data, https://ec.europa.eu/eurostat/web/education-and-training/database). As a matter of fact, the correlation between the system’s size and participation in EUi is 0.75 (excluding Turkey), showing that participation is by and large proportional to size (Fig. 3), except for the UK.

Although the UK has a large HE system, the number of participating HEIs is relatively small. After the exit from the EU in 2020, the UK has opted out from participation in the Erasmus+ programme 2021–2027 (as an associate member) and thus is currently not eligible to apply for funding from the EU and is currently setting up its own national replacement scheme (Brooks & Waters, 2023). While UK HEIs were eligible in the pilot calls, it is quite possible that uncertainty about the future of European cooperation reduces the overall interest in participating in the UK (Cavallaro & Lepori, 2021; Highman et al., 2023).

Perhaps the most useful way of looking at the geographical patterns in terms of balance is to consider *participation by a group of countries* instead. Some 69% of all members come from the so-called EU15 states (i.e. countries that participated in the EU since before 2004; here without the UK), while 23% come from the EU13 countries (new MS who joined the EU after 2004). The remaining 8% of members come from non-EU countries: the UK (9 HEIs), Norway (8), Switzerland (3), and Serbia (2).

There has been a further change leading to an even greater geographical balance between the participation of these groups of countries in 2022—40% of the *new members* come from either the EU13 states (26%) or from the non-EU countries (14%). All alliances now include EU13 countries (and/or Serbia), although, in only 19 of them, the proportion of the EU13 countries exceeds one quarter. It seems the “rules of the game” have indeed succeeded in generating a reasonably good geographical spread: firstly, the alliances must include member institutions from different parts of Europe; and, secondly, HEIs can participate only in a single alliance, and thus, the pool of prestigious HEIs is limited, generating incentives to broaden the scope geographically.

This assumption is supported by combining geographical patterns with ranking positions. While only 40% of EU15 participations include unranked HEIs, 72% of EU13 participations are those of unranked HEIs. Notably, of the non-EU participating HEIs, only 25% are unranked institutions showing that the cohesion argument is closely related to the EU perimeter (see also Online Resource 1).

The current patterns generally mirror the spread of top- vs lower-ranked institutions in Europe. Looking more closely at the countries with the highest numbers of HEIs included in the rankings (such as France, Germany, Spain, and Italy), we found a large proportion of their ranked institutions to participate in the EUi. For example, in 2021, 50 HEIs from Germany were included in the top 1000 ranks in ARWU. Of those, 26 participated in EUi (with an additional 16 participations by non-ranked HEIs). On the contrary, in Poland, only 10 HEIs featured in the top 1000 ranks, but of those, 7 already participated in EUi (with an additional 11 participations by non-ranked HEIs), and Romania had only one institution included in the top 1000—it participated in the EUi alongside 13 non-ranked HEIs.

The pattern is strikingly different in the UK and Switzerland: in both countries, only a minority of ranked institutions (UK: 9 out of 65; CH: 3 out of 10) are currently participating; in both countries, unranked institutions do not feature in the alliances at all.

### The role of pre-existing ties

**Fig. 4:**
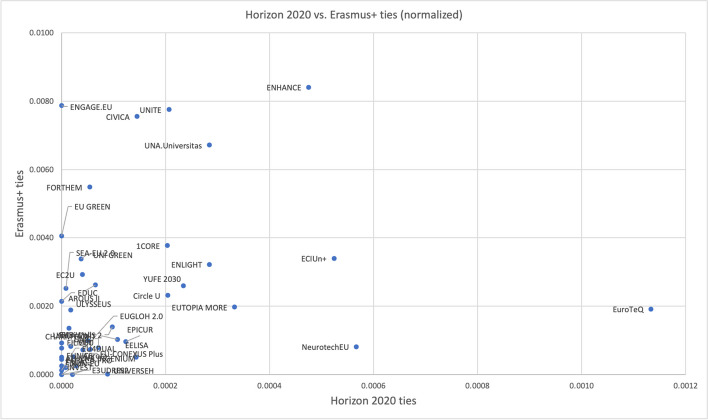
EU-FP and Erasmus students’ exchanges by alliance. X: number of joint participations to H2020 projects between pairs of HEIs belonging to an alliance normalised by the total number of students in the alliance (2014–2020). Y: number of exchanges of students between pairs of HEIs belonging to an alliance normalised by the total number of students in the alliance (2014–2020)

The analysis of previous ties in European Framework Programs and Erasmus+ provides only weak evidence of their role in the alliance’s formation. Indeed, many of the alliances had pre-existing network ties in terms of collaborations in the EU-FP Horizon 2020 (notably, however, these are researcher-initiated collaborations, affecting a research group or department rather than the whole institution). More than half had also exchanged students under the Erasmus+ programme (Fig. 4).

However, the evidence here is mixed, with few alliances having a large number of such ties in both programmes, others only in one, and many neither in EU-FPs nor in Erasmus+. For example, Unite, which originates from the Consortium Linking Universities of Science and Technology for Education and Research (CLUSTER), had many ties in both Horizon 2020 and Erasmus+, while the CIVIS 2 alliance, with most of the original (pilot phase one) HEIs being members of UNICA network of universities, has average to low number of network ties within both programmes compared to other alliances. As a median, a pair of HEIs, which are members of the same alliance, had just one EU-FP project together and exchanged 33 students over the whole period 2014–2020.

On the contrary, we identified many previous institutional cooperations within associations or institutional networks preceding the EUi among two or more member institutions within the alliances in different configurations and various arrangements, in particular, among the alliances formed under the first pilot call in 2019. Some of the alliances have evolved directly from previous networks (e.g. EuroTeQ, YUFE2030). For several others (1CORE, CHARM EIGHT, CIVIS 2, EC2U, ENHANCE), we found that all, or almost all, member HEIs have participated in the same network (which included also other HEIs). In some instances, this includes original members only; in others, at least some of the new members as well. In a few cases (UNA.Universitas, ARQUS II, EUGLOH 2.0, ENLIGHT, Circle U.), the memberships in two or more groups overlap in such a way that every alliance (original) member is linked to at least some others through those pre-existing networks. Notably, however, many of the newer alliances (formed in 2020) have no, or little previous overlapping memberships in other networks. Further, some new members who joined the existing alliances in 2022 do not seem to have previously shared institutional memberships in other networks. Finally, in the four new alliances formed in 2022, none (in EU Green) or a small proportion of members shares previous connections in other networks (detailed information is given in Online Resource 2). Overall, it appears that participation in whole-institution networks rather than research collaborations or the history of student exchanges influenced the creation of alliances within the framework of the EUi, in particular during the initial period.

In summary, our findings indicate that alliances have been principally formed between *similar HEIs* (in particular in terms of status), often *with pre-existing ties* created primarily through long-standing cooperation through other networks or alliances, confirming earlier results from a three-case study reported by Charret and Chankseliani (2022). We have demonstrated empirically that some of the policy design measures, namely the requirement for broad geographical coverage and generically framed rules for participation, have generated opportunities for participation of the less prestigious (according to the global rankings) institutions in particular from the EU13 states, in selected alliances, thus broadening the scope of the EUi beyond the core of top-ranked research universities, somewhat balancing the scales between excellence and inclusiveness. On the contrary, however, we have also found that the inclusion of non-universities HEIs remains fairly limited.

## Discussion

In this paper, we examined the diversity of participating HEIs, the composition of—as well as the mechanisms behind—forming individual alliances within the framework of the European Universities initiative. In this final section, we respond empirically to the overall question as posed by others (see in particular Birk (2019) and Gunn (2020)), discussing to what extent a balance between excellence and inclusiveness has been achieved thus far within the scheme.

It should be acknowledged that *the uptake* of the EUi has been considerable, with several hundreds of HEIs from across Europe working together to prepare submissions for the scheme—some resubmitting to a later call if not initially successful. The appeal of the EUi for the European HEIs lies likely, at least in part, in the tangible resources available now and in the future. Firstly, opportunities are afforded through sharing resources with others and gaining the “critical mass necessary to better compete globally” (Harrison et al., 2016, p. 928). The alliances are currently supported under a long(er)-term funding model than other European projects, with dedicated funds from Erasmus+ in combination with the EU-FP and other EU instruments. Many HEIs receive additional support from national governments, either through targeted funds (in 20 countries) or funds integrated into the HEI’s core funding (in 17 countries) (Jongbloed et al., 2022). The alliances are also petitioning MS and European institutions to establish a dedicated “holistic and sustainable long-term funding” model, combining resources from different existing programmes^5^ for the future. Yet, participation in the initiative also requires a commitment of own resources, and—at least in the shorter term—the financial benefits are unlikely to be the only incentive for HEIs to participate.

Indeed, not less important is the competition for intangible resources—access to policymakers and a “place at the table” when decisions about the future of European HE are made, but also *status and social recognition*, which, as discussed above, affect the interactions between HEIs and their environments (Horta et al., 2008). A conceivable explanation of the high interest in the scheme is that HEIs consider the potential strong signalling value of participation in the EUi as a *European label of excellence*, as the architects of the programme themselves refer to the alliances as the “universities of the future”, leading “in quality, performance, attractiveness and international competitiveness” (European Commission, 2022) in Europe.

Yet, not unlike the recent changes in European research funding (namely the introduction of the European Excellence Initiative within the framework of Horizon Europe), the EUi aims to *balance excellence* with the overall political aim of the European Union—*cohesion*, principally through comprehensive *geographical coverage*. One of the few formal criteria for the composition of the alliances from the beginning has been that they include HEIs from different parts of Europe, aiming to bridge the west-to-east gap in research and achieving wider territorial cohesion across the EEA and beyond. Indeed, after two pilots and one “regular” round of funding, the scheme appears to be *geographically balanced*, with participation proportional to the number of HEIs in the different parts of Europe. This can be contrasted with the uneven participation and budget distribution in EU Framework Programs, which have been thus far visibly biased against the EU13 countries (Quaglio et al., 2020). While the alliances fare quite well in terms of geographical inclusiveness, institutional inclusiveness is less evident.

We have found in this study that the scheme exhibits a limited horizontal diversity, encompassing a narrow range of higher education institutions. Only 20% of current EUi members are universities of applied sciences, technical or technological institutions and art schools, and the vast majority (92%) are PhD-awarding institutions (these are primarily research and generalist universities). There is also little evidence of mixing between the research/generalist universities and more specialised HEIs like the universities of applied sciences.

PhD-awarding institutions can be otherwise described as research-active HEIs and, as such, able to compete in the global rankings. Indeed, status (operationalised through rankings position) influences both the participation of individual HEIs and the composition of the alliances within the EUi, albeit to a lesser extent than initially expected. It seems, as shown above, the generically framed rules for participation and encouragement from the EC (including through financial incentives) to enlarge existing alliances have generated opportunities for participation of the lower-status HEIs, thus broadening the scope of the EUi beyond the core of top-ranked research universities in knowledge production centres of Europe, which made up many of the pioneer alliances in 2019. The enlargement has led not only to the creation of new alliances made up of lower-ranked HEIs but also to the extension of existing alliances to incorporate new partners (often located geographically in the EU13 countries) from the lower-status strata.

Although, as noted in the theoretical framework, higher-status institutions generally tend to collaborate with those with similar standing (Burris, 2004; Chung et al., 2000; Podolny, 1994), under certain market conditions, this tendency can be overridden (Collet & Philippe, 2014). The current market conditions for HE in Europe are mixed with a number of challenges, including funding cuts, demographic changes, and competition (for students and personnel) from foreign HEIs, but also opportunities for growth for the HEIs which are able to adapt and evolve (e.g. by teaching online, diversifying programme offer, improving mobility opportunities for students or staff, or expanding recruitment to foreign markets). Although it appears that status remains central for the time being, under current conditions, there might be reasons to enlarge to lower-status HEIs, particularly when alliances include a core of ranked institutions: As European policymaking in the areas of higher education and research moves towards capacity building and cohesion, higher-ranked institutions not wanting to miss out on this high-potential opportunity arguably risk little (Patterson et al., 2014, c.f. Podolny, 1994) by inviting lower-ranked HEIs to join them while securing the longer-term backing of the policymakers. As noted in past research, ties with new organisations offer opportunities for learning independent of status (Podolny et al., 1996; Shipilov et al., 2011) providing high-status organisations with access to novel knowledge and resources “even when the prospects of some alliances may be doubtful” (Collet & Philippe, 2014, p. 412). It appears that the promising opportunities of EUi seem to supersede other concerns, at least in some of the alliances.

From the perspective of the lower-status HEIs participating in the EUi, on the other hand, entering alliances made of higher-status partners offers potential for status growth (Podolny, 1994). However, partnering with similar (lower) status HEIs may lead to more equitable partnership while, at the same time, challenging the established hierarchies, substituting the existing status signals (such as position in the global rankings) with new ones, that is, membership in European Universities alliance.

While encouraging collaboration between HEIs in the EU13 countries with top European research organisations has been previously recommended as a policy direction for helping mitigate the innovation gap in Europe (Quaglio et al., 2020, p. 21), and institutional diversity has been encouraged by the architects of the EUi, the question of the consequential nature of the alliances remains. Future evaluations of the EUi should assess whether all members, including those from lower-status strata and those located in the EU13 countries, remain in the alliances long term, and whether the formal shared membership in an alliance by HEIs of different standing translates to more collaboration between them in practice beyond the rhetorical assurances during the application process. It will be interesting to repeat the study in late 2024, following the conclusive (at least according to current plans) enlargement of the EUi, to assess the balance between inclusion and excellence within the EUi in its intended final form. We can also foresee future studies on alliances’ governance structures examining whether status hierarchies will reproduce within the alliances, resulting in an unequal share of benefits from participation in the EUi, undermining the overarching goal of achieving cohesion across the European Union, and tipping the scales back towards excellence instead.

## Supplementary information

ESM 1 (XLSX 12 kb)

ESM 2 (XLSX 15 kb)

## Data Availability

The quantitative data analysed in this study is publicly available via sources indicated in the text of the article.

## Footnotes

1 From 2022, all institutions within the European Higher Education Area can join alliances as “associate members”.

2 Cohesion, that is the process of reducing regional disparities and promoting of balanced and harmonious social, economic, and territorial development, is one of the EU’s core values and a key objective of its policies (European Parliament, 2023).

3 Our ETER dataset in fact includes 368 entities—this is because at the time of analysis ETER included data up to academic year 2020, thus not covering some of the relevant demographic changes. For example, two Irish institutions—members of RUN-EU—have merged recently, but the new Technological University of the Shannon: Midlands Midwest has not yet been assigned a new ID in ETER; thus, in the sample, the two institutions which later merged are included. In two alliances—YUFE and ARQUS, the French member institution is a university group—ETER IDs of participating HEIs have been included in the analysis.

4 This differs from the number of HEIs involved cited in the official sources (340). This is because, at the time of analysis, ETER included data up to the academic year 2020, thus not covering some of the relevant recent demographic changes. See note no. 3 for an example. Although UK and Swiss institutions cannot be funded through the Erasmus+ and are thus formally associate partners, according to alliances’ own webpages, these HEIs (except for the University of Basel) are considered equal to full members, represented and active in all layers of governance, management, and activities of the alliances, and are therefore included in our analysis.

5 See the Call for sustainable and holistic support to European University Alliances: https://www.unite-university.eu/media/for-eu-on-funding-needs-incl-research-support_052022.pdf builds upon the previous Alliances statement on funding needs (8 March 2022).
